# The impact of social activities, social networks, social support and social relationships on the cognitive functioning of healthy older adults: a systematic review

**DOI:** 10.1186/s13643-017-0632-2

**Published:** 2017-12-19

**Authors:** Michelle E. Kelly, Hollie Duff, Sara Kelly, Joanna E. McHugh Power, Sabina Brennan, Brian A. Lawlor, David G. Loughrey

**Affiliations:** 1 Department of Psychology, School of Business, National College of Ireland, 2nd Floor, Mayor Street, IFSC, Dublin, 1 Ireland; 2 The NEIL Programme, Institute of Neuroscience, Trinity College Dublin, Dublin, 2 Ireland; 3 ADAPT Centre, Trinity College Dublin, Dublin, Ireland; 4 Global Brain Health Institute, Trinity College Dublin, Dublin, Ireland

**Keywords:** Systematic review, Meta-analysis, Social relationships, Social activity, Social engagement, Cognitive function, Executive function, Working memory, Healthy older adults

## Abstract

**Background:**

Social relationships, which are contingent on access to social networks, promote engagement in social activities and provide access to social support. These social factors have been shown to positively impact health outcomes. In the current systematic review, we offer a comprehensive overview of the impact of social activities, social networks and social support on the cognitive functioning of healthy older adults (50+) and examine the differential effects of aspects of social relationships on various cognitive domains.

**Methods:**

We followed PRISMA (Preferred Reporting Items for Systematic Reviews and Meta-Analysis) guidelines, and collated data from randomised controlled trials (RCTs), genetic and observational studies. Independent variables of interest included subjective measures of social activities, social networks, and social support, and composite measures of social relationships (CMSR). The primary outcome of interest was cognitive function divided into domains of episodic memory, semantic memory, overall memory ability, working memory, verbal fluency, reasoning, attention, processing speed, visuospatial abilities, overall executive functioning and global cognition.

**Results:**

Thirty-nine studies were included in the review; three RCTs, 34 observational studies, and two genetic studies. Evidence suggests a relationship between (1) social activity and global cognition and overall executive functioning, working memory, visuospatial abilities and processing speed but not episodic memory, verbal fluency, reasoning or attention; (2) social networks and global cognition but not episodic memory, attention or processing speed; (3) social support and global cognition and episodic memory but not attention or processing speed; and (4) CMSR and episodic memory and verbal fluency but not global cognition.

**Conclusions:**

The results support prior conclusions that there is an association between social relationships and cognitive function but the exact nature of this association remains unclear. Implications of the findings are discussed and suggestions for future research provided.

**Systematic review registration:**

PROSPERO 2012: CRD42012003248.

**Electronic supplementary material:**

The online version of this article (10.1186/s13643-017-0632-2) contains supplementary material, which is available to authorized users.

## Background

Cognitive functioning plays an important role in determining functional abilities, quality of life and independence in older adults [1, 2]. Although changes in cognitive function such as processing speed, episodic memory and executive functions are typical with normative cognitive ageing [3–5], cognitive decline is not a part of healthy ageing [6–8]. Evidence suggests that cognitive function in older adults may be affected by modifiable risk and protective factors including smoking, poor diet, levels of physical activity, cognitive stimulation and social relationships [9–13]. With an increasing ageing population, cognitive ageing researchers are prioritising exploration of these lifestyle factors as they may provide a pathway to interventions to prevent cognitive decline or maintain cognitive function in older adults. Of these lifestyle factors, social relationships are of particular interest as improving factors associated with social relationships may offer a relatively simple method of promoting positive outcomes in cognitive functioning.

One difficulty when trying to determine the effect of social relationships on cognitive function is the use of discrepant and unclear definitions of different social factors [14]. If researchers are to make accurate recommendations regarding activities that can promote cognitive health, they need to use precise terminology to ensure consistency and clarity of information presented. In an attempt to address the issue of discrepant definitions in the literature, Berkman and colleagues suggested a framework to clarify terms describing social factors and behaviours [15]. Berkman et al. explain that social integration, which includes upstream levels of social resources, community, and family, promotes access to social networks. Social networks, defined as “the web of social relationships that surrounds an individual” (p.847) in-turn facilitate engagement in social activities and access to social support [15]. Social relationships therefore, are both impacted by and influence social networks, social activity and social support [15–17]. Social network characteristics can include the network size, the relationship between members of the network, and the frequency of contact between network members [15]. Examples of social activity, also known as social participation or engagement, may include meeting friends, attending events or functions, volunteering or participating in occupational duties or group recreational activities [15]. Social support, often divided into emotional, instrumental, and informational support refers to a person’s perception of the availability of help or support from others in their social network [15]. In two recent reviews, Kuiper and colleagues also refer to social relationships and explain that social networks and activity represent structural aspects of social relationships, while social support represents functional aspects of social relationships [18, 19]. In the interest of promoting the consistent use of well-defined terminology across reviews, we will use the terms as presented by Berkman et al. and Kuiper et al., and refer to the overall category of social relationships which incorporates factors including social networks, activity and support.

Research examining the effects of social relationships on older adults’ cognitive functioning most commonly assesses the frequency of engagement in social activities [20], followed by social network size/structure [21] and social support [22]. Cross-sectional studies examining the relationship between engagement in social activity and cognitive function have found that an active and socially engaged lifestyle is related to improved cognitive function in ageing [23, 24]. Results from observational studies investigating longitudinal associations between social relationships and older adults’ cognitive function are not conclusive however [25]. While many observational studies have found that aspects of social relationships, such as social activity, are related to benefits in cognitive function [9, 17, 21, 26–29], others have failed to do so [30–33]. The lack of consistency across observational studies is further compounded by a lack of supportive evidence from randomised controlled trials (RCTs). In their literature review, Wang et al. searched for RCTs to supplement correlational data on social activity and cognitive function, but found none, thereby limiting the extent to which causal relationships could be inferred [2]. Since then, to our knowledge, no RCTs examining lifestyle factors and cognitive function have included any social factors as primary intervention targets. Perhaps this lack of RCTs results from difficulties translating aspects of social relationships into a measurable experimental design [34]. Nonetheless, some RCTs have included engagement in social activities as active control conditions [34, 35], but these have yet to be examined in the context of a systematic review.

Consideration should be given to possible effects that different aspects of social relationships may have on specific cognitive processes [36–38], as each unique outcome may result in differential effects on the trajectory of cognitive decline [39]. The literature has previously been criticised for not permitting an assessment of domain-specific effects of social relationships on cognition [36]. In response to this, Gow et al. examined the effects of social relationship factors on general cognitive ability, memory and processing speed and found that more social support was associated with better general cognitive abilities but not memory, and less social support was associated with slower processing speed; these associations were partly accounted for by symptoms of depression. Other studies have also reported that different aspects of social relationships can differentially impact cognitive functioning, and discrepancies between these factors can have clinically meaningful effects on cognitive function [19, 40] and incident dementia outcomes [18].

From a theoretical perspective, there is clear reason to expect an association between social relationship factors and cognitive outcomes. Social activities may include a type of cognitive stimulation, thus contributing to cognitive outcomes via the building of cognitive reserve [41]; cognitive reserve optimises cognitive performance through the recruitment of alternate brain networks and cognitive strategies to compensate for cognitive difficulties related to pathology [42, 43]. Social support in particular may impact cognitive outcomes via its buffering effect on stress [44]. Interacting with others in one’s social network may influence cognitive outcomes because close social ties makes positive health behaviours more likely (social control hypothesis; [45]). An intriguing alternative hypothesis has been put forward by Adolphs who proposes that all cognition is intrinsically social in nature [46]. It is also reasonable to assume that different aspects of social relationships may affect cognitive domains in different ways. For example, reducing stress through social support is likely to benefit memory and executive functioning [47, 48], while social network interactions may indirectly benefit reasoning, attention and processing speed through encouraging health behaviours such as exercise [12, 49]. A detailed investigated of how social relationships may affect different cognitive domains has yet to be conducted.

Few literature reviews provide a comprehensive overview of research on social relationships and cognitive function in older adults. Structural and functional aspects social relationships are not often differentiated or separately assessed [50], precluding any meaningful comment on their distinct contributions towards cognitive ageing. In addition, social factors tend to be considered alongside or as part of other lifestyle factors such as leisure or intellectual activity [41]; outcomes such as mortality, physical health, or dementia are included in lieu of cognitive function [10, 18]; only specific aspects of social relationships are considered, such as social isolation [51]; or reviews do not include comprehensive systematic literature searches [52, 53]. The findings from prior reviews have shown a positive effect of engagement in social activities on verbal fluency [53] and of ‘socialisation’ on overall cognitive function [54], but findings were inconclusive and terminology used was inconsistent. Two more recent reviews that used consistent, well-defined terminology and examined various aspects of social relationships found that poor social relationships were associated with an increased risk of cognitive decline [19] and dementia [18]. A comprehensive review has yet to be conducted that clarifies the effect of social relationships on the cognitive function (continuous outcome) of older adults.

**Fig. 1:**
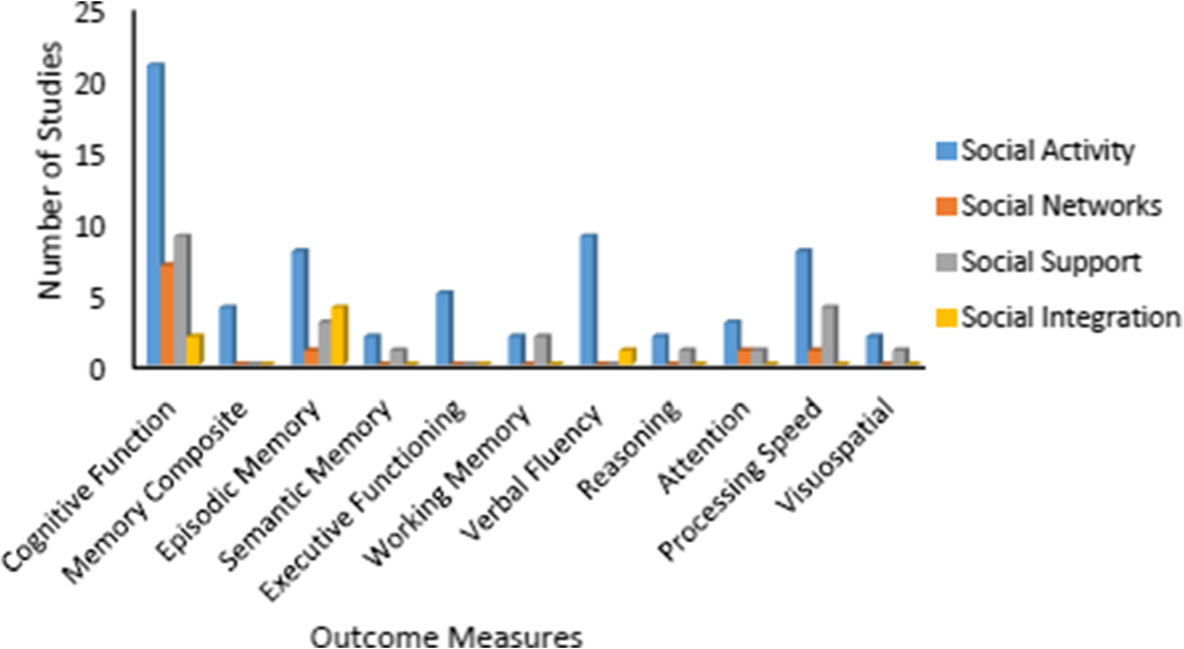
The graph shows the total number of studies (y-axis) that included outcome measures to assess cognitive abilities (x-axis) for each of the four categories of social engagement (figure key)

The aim of the current review was to evaluate the association between different aspects of social relationships; specifically social activity, social networks, and social support, with the cognitive functioning of healthy older adults with no known cognitive impairment. This is the first review to include data from available RCTs and genetic (twin) studies alongside an updated summary of observational evidence. The review is also innovative in its attempt to account for the differential effects of various factors associated with social relationships on specific cognitive domains (Fig. 1).

## Methods

### Search strategy

**Fig. 2:**
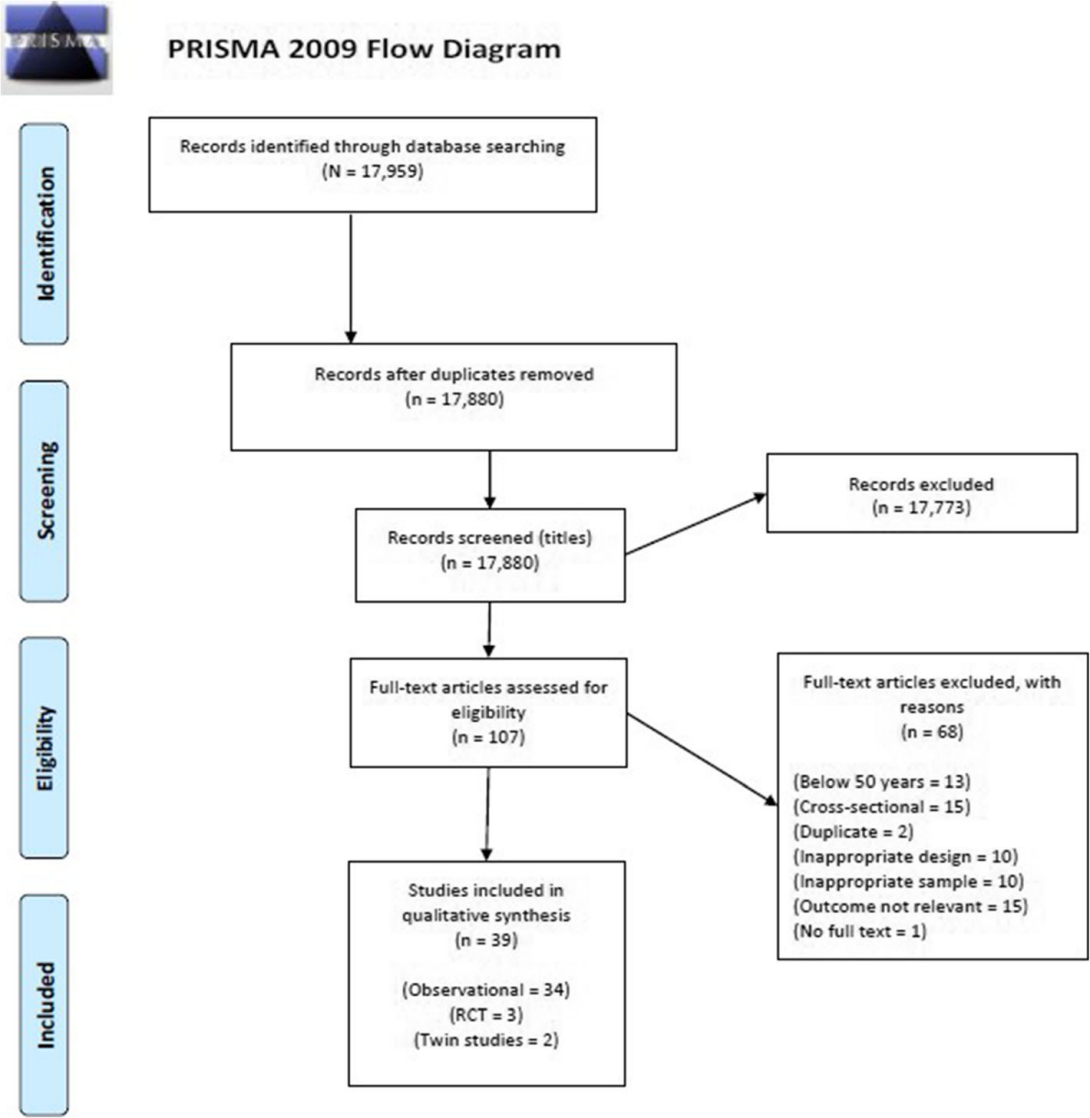
PRISMA flow diagram

PubMed, Medline and PsycInfo were searched to identify RCTs, observational, and twin studies written in English and published between January 2000 and January 2017. The original search was conducted in 2015, and was updated in 2017. Search terms included ‘social activity’, ‘social engagement’, ‘social intervention’, ‘leisure intervention’, combined with ‘cognition’, ‘cognitive performance’, ‘cognitive decline’, ‘cognitive function’ and ‘healthy elderly’, ‘older adults’ (see Additional file 1 for full search strategy). Database searches were supplemented by searches of Google Scholar and hand searches of the reference sections of relevant reviews and included studies. Titles and abstracts were screened to exclude articles that did not meet inclusion criteria. Full texts of remaining studies were then screened for eligibility by two independent reviewers. Disagreements were resolved through discussions with the review team (see Fig. 2). This review is part of a series of reviews aimed at examining the impact of non-pharmacological interventions of the cognitive functioning of healthy older adults [12, 13, 56]. The review series was registered with the PROSPERO International prospective register of systematic reviews in 2012 (CRD42012003248).

### Selection criteria

We followed Preferred Reporting Items for Systematic Reviews and Meta-Analysis (PRISMA) guidelines. The following inclusion criteria were used: (1) peer-reviewed and academically published observational, RCT or twin studies that (2) investigated the impact of engagement in social activities, social networks or social support on cognitive function and (3) included a sample of community dwelling older adults (> 50 years) with no known cognitive impairment. We excluded studies if participants had been diagnosed with any cognitive impairment, cardiovascular disease, or other significant medical, psychiatric or neurological problems; or studies that combined data from participants with cognitive function within the normal range with data from participants experiencing cognitive decline (see excluded studies table, Additional file 2).

### Outcomes of interest

The primary outcome of interest was cognitive function. In line with a Cochrane review [55] and three prior reviews published by the review team [12, 13, 56], cognitive outcome measures were grouped into separate ability subgroups within the cognitive domains of memory and executive function. This permitted the comparison of data that was as homogenous as possible. Within the memory domain, outcomes were categorised to include measures of episodic memory, semantic memory or overall memory ability (measured by global or composite measures of memory). Within the executive function domain, outcomes were categorised to include measures of working memory, verbal fluency, reasoning, attention, processing speed, visuospatial abilities or overall executive functioning (measured by global or composite measures of executive function). Global cognition was measured using global or composite measures of cognitive function.

Social relationships were categorised based on the suggested framework of Berkman et al. [15] and included social activity, social networks, social support and composite measures of social relationships (CMSR). *Social activity* included engagement in facilitator led group discussions, social interactions, field trips, travel or outings, visiting and receiving visitors, participation in voluntary activities, religious activities, membership in community groups or associations, or attending social groups. *Social networks* included living arrangements, marital status, number of social ties or frequency of contact with friends and family. *Social support* included emotional support, satisfaction with support, positive or negative interactions, instrumental support, informational support, someone to share personal experiences and feelings with, help with decision making, support with daily tasks and general ratings of social support. Any combination of social activity, social networks and/or social support measures were considered as CMSR.

### Data extraction

Data related to our outcomes of interest were extracted by two independent reviewers and cross-checked by an expert author. Cross-sectional and longitudinal outcomes were recorded that examined the relationship between social relationships and cognitive function. Multiple publication bias was avoided by using data from the most recently published study. Where two studies used data from the same cohort but presented different relevant outcomes, both were included on the same cell of the included studies table. Priority was given to outcomes that were adjusted for covariates/controlled for potential confounds. Due to large diversity between study’ definitions and measurement of social relationships, cognitive outcomes measured, and analysis used, and in line with recommendations of from Section 9 of the Cochrane handbook, meta-analysis was not conducted as it was unlikely to derive meaningful conclusions. Guidelines from Section 8 of the Cochrane Handbook were used to assess risk of bias in RCTs (Additional file 3). The STROBE assessment tool was used to assess the quality of reporting in cohort studies (Additional file 4).

## Results

Thirty-nine studies were identified that met the inclusion criteria outlined above; three RCTs including 576 participants, 34 observational studies including 87,509 participants in 32 longitudinal data sets, and two twin studies including 189 pairs of participants. *Social activity* was examined in three RCTs, 22 observational studies and two genetic studies; *social networks* and *social support* were examined in nine observational studies each, and CMSR were included in three observational studies. See Tables 1, 2, and 3. Social activity was the most common type of social relationship assessed and global/composite measures of cognitive function were the most common cognitive outcome measure employed. Overall, the type of social relationship and cognitive outcome measures included varied largely across studies (see Fig. 1).

**Table 1:** Characteristics of studies: intervention studies

Intervention studies examining the impact of social relationships on cognitive function					
Study	Participants	Intervention	Socialisation defined	Cognitive outcome measures	Results summary
Mortimer (2012) [35] China	*N* = 120 30/group Age:60–79	1. Tai Chi 2. Walking 3. *Social activity* 4. No intervention 3 times/week, 40 weeks RCT	Social activity: Meeting and conversational discussion facilitated by leader and assistant 3 times a week for 40 weeks.	Memory (composite AVLT, CVLT) Attention (Bell Cancellation Test, Stroop, TMT A) *Verbal Fluency (Category)* ^*a*^ Executive function (Rey CFT) Processing speed (WAIS Digit Span, WAIS Similarities) Cognitive function (Boston Naming Test, Clock-Drawing Test, MDRS)	Baseline to 40-week follow-up; improved verbal fluency (*p* = 0.01), trends for improvement (*p* < 0.10) on TMT A and AVLT. Increased brain volume in the social interaction group versus no intervention, (*p* < 0.05). *P*-values not provided for other comparisons.
Park (2014) [34] USA	*N* = 221 1. *n* = 29 2. *n* = 35 3*. n* = 42 4. *n* = 36 5. *n* = 39 6. *n* = 40 Age: 60–90	Cognitive Engagement 1. Photo group 2. Quilt group 3. Dual photo + quilt control group 4. Social activity 5. Placebo 6. No intervention 15.9 h/week, 14 week programme, Non-RCT	Social activity: Participants engaged in on-site, facilitator-led social interactions, field trips, and entertainment with a social group.	Episodic memory (Cantab, HVLT) Visuospatial processing (Cantab, Raven’s Progressive Matrices) Processing speed (Digit Span) Attention/inhibitory control (Flanker Task) Cognitive function (MMSE)	Social group showed greater, but non-significant pre-post-test improvements versus photo, quilt and placebo (*p* = 0.10) on processing speed; photo and placebo on attention/inhibitory control; placebo on episodic memory; and quilt, dual and placebo on visuospatial processing.
Pitkala (2011) [57] Finland	*N* = 235 1. *n* = 117 2. *n* = 118 Age: 75+	1. *Social activity* plus therapeutic writing/group exercise/art experience 2. Normal community care 6 h/week, 3 months 12 month FU, RCT	Social activity: Choice of 1/3 activities plus active discussions, shared experiences, discussed feelings, peer support. Facilitated by trained professionals.	*Cognitive function (ADAS-Cog)* ^*b*^ *Subjective Cognitive Function (15D)* ^*b*^	ADAS-Cog scores improved significantly more in the social group than in the control group (*p* = 0.023); as did changes in 15D (*p* = 0.047).

*RCT* randomised controlled trial, *FU* follow-up, *AVLT* Auditory Verbal Learning Test, *CVLT* Category Verbal Fluency Test, *TMT* Trail Making Test, *Rey CFT* Rey Complex Figure Task, *WAIS-R* Wechsler Adult Intelligence Scale-Revised, *MDRS* Mattis Dementia Rating Scale, *Cantab* Cambridge Tests of Cognitive Function, *HVLT* Hopkins Verbal Learning Test, *MMSE* Mini Mental Status Examination, *ADAS-Cog* Alzheimer’s disease Assessment Scale-Cognition

Italic text indicates factors that were significantly related

^a^Significant improvement reported from baseline to follow-up

^b^Significant improvement reported in intervention compared to control

**Table 2:** Characteristics of studies: observational longitudinal studies

Observational longitudinal studies examining the impact of social relationships on cognitive function					
Study	Participant	Years FU	Social activity defined/measure	Cognitive outcome measures	Results
Aartsen (2002) [30] Netherlands (LAS)	*N* = 2076 Age: 55–85 M Age = 68.7	6 years	Social activity: activities, social status, and service, incl. Visiting church, visiting neighbourhood associations, attending organisation for helping older adults, neighbours, or disabled persons.	Cognitive function (MMSE) Episodic Memory (Immediate Recall, AVLT: 15 Words Test) Reasoning (Raven’s Coloured Progressive Matrices) Processing speed (Coding Task)	No association between indicators of social activity and cognitive functioning.
Andrew (2010) [78] Canada (CSHA)	*N* = 2468 Age: 70+ M Age = 79.1	5 years	*Social support*: self-report potential social deficits - living situation, social support, family relationships, friendships, etc.	*Cognitive function* (Modified MMSE: 3MS) ^c^	Each added baseline social deficit was associated with increased odds of cognitive decline (OR, 1.03; 95% CI, 1.00 to 1.06; *p* = .02, *N* = 2391). Compared to low social vulnerability, high social vulnerability led to 36% increased odds of cognitive decline (OR, 1.36; 95% CI, 1.06 to 1.74).
Barnes (2004) [17] USA (CHAP)	*N* = 6102 Age: 65+	5.2 years	*Social networks:* number of children, relatives, and friends seen at least once a month. *Social activity:* 4 items related to social and productive activity.	*Cognitive function* (composite including measures of **e**pisodic memory, perceptual speed, and MMSE) ^c^	Greater social networks and social activity positively correlated with initial level of cognitive function (both *p* < 0.001). Rate of global cognitive decline reduced by 39% for 16 social ties versus 1 social tie; and reduced by 91% for a max score of 8 versus a score of 0 on the social engagement (activity) scale.
Beland (2005) [61]; Zunzunegui (2003) [74] Spain (Ageing in Legane’s)	*N =* 1571 Age: 65–100 M Age = 75.6	7 years	*Social activity:* frequency of attending community associations, religious services, recreational activities. *Social network:* no. relatives seen monthly, presence of friends *Social support:* how often helped children, family, and friends; or felt useful or important to them.	*Cognitive function (*Leganes’ Cognitive Function Test; PCL**:** composite of orientation and memory items from Short Portable Mental Status Questionnaire, the Barcelona Test and EPESE short story recall**)** ^b^	Social activities, network, and support were associated with rate of change in cognitive function (coefficient equal to or greater than twice the standard error, no *p*-values provided). Those with greater social networks and support (compared to those with less) maintained better cognitive functioning up to 80 years. (Beland, 2005). Social network (visual contact with family, *p* = 0.028) and activity (*p* = 0.040) were significant predictors of cognitive decline for both sexes at follow-up. Engagement with friends predicted lower probability of decline for women (*p* = 0.013) (Zunzunegui, 2003).
Bielak (2007) [20] Canada (VLS)	*N* = 530 Age: 55–94 M Age = 68.5	2 years	*Social activity:* frequency of engagement in social activities incl. Attending concerts or visiting friends. VLS Activity Lifestyle Questionnaire	Executive functioning (*Lexical decision making* ^*b*^ *;* semantic decision making; simple and choice reaction time)	Social activities associated with lexical decision making only (*p* < 0.05).
Bourassa (2017) [72] EU (SHARE)	*N = 19,832* Age: 50+ M Age = 64.4	6 years	*Social activity:* frequency of participation in volunteer work, social club, religious organisation, community/political group.	*Episodic Memory* (Ten Word Delayed Recall Test; immediate and delayed recall) ^c^ *Verbal fluency* (Category Fluency Task) ^c^	Higher levels of social activity associated with higher memory and fluency scores at BL (*p* < 0.01), time 1 (*p* < 0.01) and time 3 (*p* < 0.05); and social activity at baseline predicted change in memory and fluency over time (*p* < 0.01). Replicated in a second subsample.
Bosma (2002) [62] Netherlands (MAAS)	*N* = 830 Age: 49–81	3 years	*Social activity:* frequency per week of engagement in organisational memberships, e.g. clubs.	Episodic Memory (Verbal Learning Test - immediate and *delayed recall* ^*b*^ *, total recall* ^*b*^ *)* Attention (Stroop Test) Processing speed (Letter-Digit Coding Test) Verbal fluency (Word Fluency Test) Cognitive Function (MMSE)	Compared to no social activities, socially active participants scores decreased 0.94 points less on total recall (*p* < 0.05) and 0.30 points less on delayed recall (*p* < 0.05).
Chen (2016) [58] Taiwan (TLSA)	*N* = 2300 Age: 60+ M Age = 70.9	14 years	*Social activity:* playing games and socialising with friends, neighbours, relatives. *Social support* (emotional support): being cared for, being listened to by friends, relatives.	*Cognitive function* (SPMSQ; short version; 5 questions) ^c^	Activity and support related to progression of decline in cognitive function. Low/declining, high/declining or high/stable groups differed in ratings of activity and support (both *p* < 0.001); lower ratings of activity and support for low/declining. A 1-point increase in emotional support decreased the odds in being in the *low/declining* group: OR, 0.77; 95% CI, 0.60 to 0.99.
De Frias (2014) [73] Canada (VLS)	*N* = 501 Age: 53–90 M Age = 68.2	4.5 years	Social activity: activities sub-score of VLS Activity Lifestyle Questionnaire	Processing speed (WAIS-R DSS) Inductive reasoning (Letter Series Test) Episodic memory (Immediate recall) Verbal fluency (ETS-CA; ETS-Recognition Vocabulary) Executive function (composite of Hayling Sentence Completion Test; Stroop; Brixton Test; Colour Trails; Computational span; Reading span)	Social activity not associated with maintenance of cognitive status (high, normal or low) over time. Social activity moderated the relationship between cognitive status and executive functioning over time but not significantly.
Ellwardt (2013) [22] Amsterdam (LASA)	*N* = 2255 Age: 55–85 M Age = 63	6 years	Social support: 9 most frequent regular socially active contacts; *Emotional support:* self-reported; *Instrumental support:* self-reported. Mediating variable: Loneliness	Processing speed (Coding Task) ^a^ *Reasoning* (Raven’s Coloured Progressive Matrices) ^a^ *Cognitive function* (MMSE) ^a^ *Composite of cognitive function* (Coding task, Raven’s, MMSE)^b^	Emotional support correlated with improved MMSE, coding (*p* < 0.001) and Raven’s (*p* < 0.01) at baseline. Emotional support directly (*p* = 0.06) and indirectly (*p* < 0.05) associated with improved cognitive function. Instrumental support only indirectly associated with cognitive function (*p* < 0.05).
Ertel (2008) [27] USA (HRS Study)	*N* = 16,638 Age: 50+ M Age = 64.5	6 years	*CMSR:* marital status, volunteer activities, and contact with parents, children, neighbours.	*Episodic Memory* (immediate recall, delayed recall) ^b^ Cognitive Status (TICS)	Higher baseline level of social relationships associated with slower rates of memory decline over 6 years (*p* < .01). Memory decline among the most socially integrated was less than half the rate of change of the least socially integrated (*p* < .01).
Glei (2005) [63]; Hsu (2007) [64] Taiwan (The SHLSE)	*N* = 2387 Age = 60+	7 years	Social network: marital status, close relatives, other relatives, friends and neighbours (all weekly contact). *Social activities*: games, socialising, organised groups, *political group; unpaid work,* religious groups, business associations, political groups, clan associations, elderly association (score 0/1–2/ 3+).	*Cognitive function* (SPMSQ**)** ^b^	No social network measure was related to cognitive function. Compared to those with 0 social activities; those with 1–2 social activities failed 13% fewer cognitive tasks (*p* < 0.001); and those with 3+ failed 33% fewer cognitive tasks (*p* < 0.001) at FU (Glei, 2005). Men in political groups at baseline were less likely to show impaired cognitive function (OR = 0.536, *p* < 0.05) Compared to no work, women with unpaid work at baseline were more likely to have impaired cognitive function at follow-up (OR = 1.652; *p* < 0.05) (Hsu, 2007)
Ho (2001) [75] Hong Kong	*N* = 988 Age: 70+ M Age = 77.4	3 years	*Social network:* (composite) contact with friends, family, neighbours; participation in community religious activities (questions adopted from the Lubben 1998 Social Network Scale). *Marital status* *Residence (community/institution)*	*Cognitive function* (CAPE) ^b^	Male participants; being divorced (*p* < 0.01), having poor social support (*p* < 0.001) and institutional living (*p* < 0.001) was associated with a greater risk of cognitive impairment (CI) at FU. Men and woman residing in institutions had 4.4 and 2.5-times increased risk of having CI compared with those living in the community.
Holtzman (2004) [21] Baltimore (ECA; Waves 1–3)	*N* = 354 Age: 50+ M Age = 61.3	12 years	*Social network:* no. of friends, relatives, neighbours *Social activity:* frequency of contact with plus no. of friends, neighbours, relatives *Social support: Emotional support*	*Cognitive function* (MMSE) ^b^	Maintenance of MMSE scores at FU were associated with baseline social network size (*p* = 0.02), and network size change (*p* = 0.02). Significant predictors of MMSE scores included network size (*p* = 0.004), social activity (*p* < 0.006), emotional support (*p* < 0.005). Frequency of contact alone did not significantly predict MMSE scores (*p* > 0.051)^1^
Hughes (2008) [76] USA (Charlotte Co Healthy Ageing Study)	*N* = 217 M Age = 72.4	5 years	Social network: family and friends Social support: emotional support, informational support; instrumental support; *satisfaction with support;* and *negative social interactions.*	*Attention* (Stroop Test) ^a^ *Processing speed* (TMT A and B) ^a^ *Episodic memory* (Hopkins Verbal Learning Test) ^b^ *Cognitive function* (Modified MMSE) ^a^	More negative social interactions (*p* = 0.03) and greater satisfaction with support (*p* = 0.02) were associated with better MMSE scores at baseline; better performance on speed and attention was associated with greater satisfaction with support (*p* = 0.01) at baseline. Over 5 years, less satisfaction with support was marginally associated with decline in episodic memory performance (*p* = 0.06).
Iwasa (2012) [65] Japan	*N* = 567 Age: 70–84 M Age = 75.8	5 years	Social activity: composite score including volunteering and group activities for the elderly.	Cognitive function (MMSE)	No significant association between social activity and cognitive decline (*p* = .14). The proportion of those with decline who did not engage in social activities was similar to the proportion of those with decline who did (20.5% vs. 17.1%, *p* = 0.31).
James (2011) [66] USA (Rush Memory and Ageing Project)	*N* = 1138 M Age = 79.6	5.2 years	*Social activity:* composite score including frequency of engagement in six common activities that involve social interaction within the last year (e.g. going out, day trips, volunteer work, visit friends, participate in groups, attend church).	*Cognitive function* (composite score from 19 tests) ^c^. *Episodic memory* (Logical Memory, East Boston Story, Word List Memory, Word List Recall, Word List Recognition) ^c^ *Semantic memory* (Boston Naming, Verbal Fluency, reading test) ^b^ *Working Memory* (Digit Span Forward and Backward, Digit Ordering) ^b^ *Processing speed* (SDMT, Number Comparison, Stroop Test) ^b^ *Visuospatial abilities* (Line Orientation, Standard Progressive Matrices) ^b^	Social activity was associated with higher baseline levels of global cognition (*p* = 0.002) and with a reduced rate of cognitive decline (*p* = 0.001) (adjusted model). A one-point increase in social activity score was associated with a 47% decrease in the annual rate of decline in global cognitive function. Social activity was associated with episodic memory at baseline (*p* < 0.001); and with reduced cognitive decline across all five domains at FU (episodic memory, *p* < 0.001; semantic memory, *p* < 0.001; working memory, *p* < 0.001; perceptual speed, *p* < 0.001; visuospatial, *p* = 0.003).
Kimura (2016) Japan (Taketoyo Project)	*N* = 100 M Age = 73.9	3 years	Social activity: *Frequency of going out* and contact with friends Social network: number of companions and *number of friends to engage in activities with*	*Cognitive function* **(**Brief Cognitive Function Examination**)** ^b^	Those with a lower frequency of going out (OR: 0.56, 95% CI, 0.80–6.38, *p* < 0.05) and fewer friends to engage in activities with (OR: 0.34, 95% CI, 0.13–0.93, *p* < 0.05) were more likely to experience cognitive decline at follow-up.
Lee (2009) [68] Korea (SLAS)	*N* = 977 Age: 65+	2 years	*Social activity:* frequency of meeting friends, neighbours, relatives; attending church; going to the movies, sports or cultural exhibits.	*Cognitive function* (MMSE - Korean version) ^b^	Social activity was significantly associated with improved cognitive scores and predicted change in cognitive function over time (*p* < 0.01).
Lee (2016) [69] Korea (KLoSA)	*N* = 1568 Age:65+ M Age = 71.1	4 years	*Social activity:* frequency of participation in church, *senior citizen’s clubs,* societies; frequency of *phone/face-to-face contact with children,* friends, family.	*Cognitive function* (MMSE - Korean version) ^b^	Those who participated in senior citizen’s clubs had a lower risk of cognitive decline at follow-up (*p* = 0.012). For those >75 years, more frequent contact with children by phone (*p* = 0.038) or face-to-face (*p* = 0.001) was associated with reduced cognitive decline.
Lövdén (2005) [59] Germany (BASE)	*N* = 516 Age: 70–103 M Age = 85	2 years	*Social activity:* Yesterday Interview (incl. Leisure, instrumental, and social activities and work). Activity List (engagement in 12 activities, incl. Restaurants, day trips, hobbies, volunteer work, travelling, etc).	*Processing speed* (Digit Letter and Identical Pictures) ^b^	Higher levels of social participation predicted a 2-year positive deviation from the average linear population decline in processing speed (*p* < 0.05); prior scores of processing speed did not modify socialisation scores.
Marioni (2014) UK (Paquid)	*N* = 3653 Age: 65+	20 years	*Social activity:* membership of a group or association, visits from family and friends, membership of a golden age club, and membership of another club.	*Cognitive function* (MMSE)^a^	The odds of being in the *high* baseline cognition group (MMSE >27) compared to the *immediate decliners* (steep linear decline) or *slow decliners* (>27 MMSE up to age 75 then steep decline) or *low* baseline cognition (<26) groups were around 10, 3, and 5 times greater for those with high social engagement.
Mousavi-Nasab (2014) [71] Sweden (Betula Project)	*N =* 794 Age: 65–85	10 years	*Social activity*: Visiting family/friends, travelling, going to a restaurant, movies, concerts, or theatre.	*Memory (*composite of free and cued recall tasks, and recognition tasks) ^b^ Verbal fluency (composite of three verbal fluency tasks and a vocabulary test)	Social activity at time 1 and time 2 significantly predicted change in memory performance at time 2 (*p* < 0.001) and time 3 (*p* < 0.001).
Nelson (2013) [81] USA (HRS-ss)	*N* = 203 Age: 50+	12 years	CMSR: marital status, volunteer activities, and contact with parents, children, neighbours.	Cognitive function (TICS) Episodic Memory (immediate and delayed recall of word list)	No significant association (adjusted analysis) between CMSR scores and cognitive function (*p* = 0.06) or memory (*p* = 0.43).
Plehn (2004) [60] Virginia, USA	*N* = 133 Age: 55+ M age = 75.6	4 years	*Social activity:* Social activities sub-score of SELF-scale	*Cognitive function* (MMSE, MDRS – Initiation and Perseveration Subtest) ^c^ *Memory* (composite, Fuld Object Memory Evaluation) ^a^ *Working memory* (Clock Drawing Test) ^a^ *Verbal fluency* (WAIS-R Vocabulary) ^a^	At baseline all measures were significantly correlated with social activity (*p* < 0.05 for clock drawing; *p* < 0.01 for vocab; *p* < 0.001 for all other measures). Participants with cognitive decline at FU reported significantly fewer baseline social activities compared to those who did not decline (*p* < 0.001).
Seeman (2001) [32] USA (MSSA)	*N* = 1189 Age: 70–79	7.5 years	Social networks: no. close social ties; no. of groups; *marital status.* Social support: *frequency of emotional support;* instrumental support; *conflicts/ demands;* support provided to others (MSSA battery).	*Cognitive function* (composite score: Boston Naming Test, WAIS-R Similarities, Copying Task, delayed spatial recognition, incidental recall, delayed recall)^c^	At baseline, marital status (p = 0.03), emotional support (p = 0.002), and conflicts/demands (*p* = 0.03) were significantly related to better cognitive functioning. Only frequency of emotional support was independently related to change in cognitive performance over 7.5 years (*p* = 0.05).
Shankar (2013) [80] UK (ELSA)	*N* = 6034 Age: 50+ M age = 65.6	4 years	*CMSR:* married/cohabiting, frequency of contact with children/immediate family/ friends and participation in organisations, religious groups, gyms, committees. UCLA Loneliness Scale (short form)	*Episodic Memory (Immediate recall of word lists)* ^*c*^ *Episodic Memory (Delayed recall of word lists)* ^*c*^ *Verbal fluency (animals)* ^*c*^	At baseline, less integration (isolation) was associated with poorer verbal fluency (*p* < 0.001), immediate recall (*p* < 0.001), delayed recall (*p* < 0.001); and loneliness was associated with poorer immediate recall (*p* < 0.001) and delayed recall (*p* = 0.02). After 4 years, increasing isolation was associated with lower scores on verbal fluency (*p* < 0.05), immediate recall (*p* < 0.001), and delayed recall (*p* < 0.001).
Small (2012) [38] Canada (VLS)	*N* = 952 Age: 55–85	12 years	*Social activity:* Frequency of engagement in social activities over the past 2 years. VLS Activity Lifestyle Questionnaire	*Verbal fluency* (lexical and semantic decision task) ^b^ Episodic memory (story recall) Semantic memory (fast recall)	Higher social activity was related to greater losses in verbal fluency (*p* < 0.05). Lower episodic memory scores were related to greater declines in social activities (*p* < .01) Lower semantic memory scores led to greater declines in social activities (*p* = 0.002).
Tomioka (2016) Japan	*N* = 6093 Age: 65+ M Age=	3 years	*Social activity:* participation in *neighbourhood associations, hobby groups, event groups* **,** senior citizen’s clubs, *volunteer activities.*	*Cognitive function* (Cognitive Performance Scale) ^b^	Relationship between more participation in social groups and lower decline for women (*p* = 0.026) but not men. 3+ activities associated with prevention of decline (OR = 0.67). Neighbourhood associations (OR = 0.81); event (OR = 0.79), hobby (OR = 0.70) and volunteer (OR = 0.66) groups, all significantly associated with lower risk of cognitive decline.
Wang (2013) [24] China	*N* = 1463 Age: 65+ M Age = 71	2.4 years	*Social activity:* Frequency of engagement in social activities from predefined list (visiting family or friends, receiving visitors at home, giving advice).	*Cognitive function* (CSID) ^b^ Episodic memory (Word List Learning; Word List Recall; IU Story Recall) Verbal fluency (Animal Fluency Test) Executive function (IU Token Test)	High level of social activity was associated with less decline in global cognition (*p* < .05), but not with episodic memory, language or executive function.
Wilson (2015) [77] USA (Rush Memory and Ageing Project)	*N* = 529 Age: 50+	4.8 years	*Social support - negative social interactions:* frequency of negative interactions (NIs), 12 items incl. Neglect/rejection, unwanted intrusion, failure by others to help, insensitive behaviour by others	*Cognitive function* (composite score on 19 tests) ^c^. *Episodic memory* ^*b*^ *Semantic memory* ^*b*^ *Working memory* ^*c*^ *Processing speed* ^*b*^ *Visuospatial abilities* ^*c*^ See James et al. (2011) above for full list of tests.	Higher frequency of NIs at baseline was associated with greater risk of MCI (HR = 1.53, 95% CI = 1.13–2.07). Higher NI score at BL associated with lower BL cognitive test score (*p* = 0.005), but not rate of decline. Higher mean NI score related to lower global cognition score (p < 0.001) and faster decline (*p* = 0.002). Higher mean NI score associated with lower level of function in all domains and faster decline in episodic, semantic and working memory.
Windsor (2014) [79] Australia (PATH)	*N =* 2551 Age: 60–64	8 years	Social support: *positive*/negative *exchanges* with family, friends and spouses.	*Episodic memory* (CVLT immediate recall) ^b^ *Working memory* (WMS-DSB) ^b^ *Processing speed* (SDMT) ^b^	More (compared to fewer) positive exchanges with friends associated with better memory (*p* < 0.05); less decline in speed (*p* < 0.05). More (compared to less) frequent positive exchanges with family associated with a slower rate of decline in speed (*p* < 0.05) Better working memory associated with increased positive exchanges with spouse (*p* < 0.05).

*MMSE* Mini Mental Status Examination, *AVLT* Auditory Verbal Learning Test, *M Age* mean age, *WAIS-R* Wechsler Adult Intelligence Scale-Revised, *DSS* Digit Symbol Substitution, *ETS-CA* Educational Testing Service Kit-Controlled Associations Test, *TICS* Telephone Interview for Cognitive Status, *CAPE* Clifton Assessment Procedure for the Elderly, *FU* follow-up, *SPMSQ* Short Portable Mental Status Questionnaire, *TMT* Trail Making Test, *SDMT* Symbol-Digit Modalities Test, *MDRS* Mattis Dementia Rating Scale, *CSID* Community Screening Instrument for Dementia, *CVLT* California Verbal Learning Test, *WMS* Wechsler Memory Scale, *DSF* Digit Span Forward, *DSB* Digit Span backward, *EPESE* Established populations for epidemiologic studies of the elderly: Resource data book, *LAS* Longitudinal Ageing Study, *CSHA* Canadian Study of Health and Ageing, *CHAP* Chicago Health and Ageing Project, *VLS* Victoria Longitudinal Study, *SHARE* Survey of Health Ageing and Retirement in Europe, *TLSA* Taiwan Longitudinal Study on Ageing, *MAAS* Maastricht Ageing Study, *LASA* Longitudinal Ageing Study Amsterdam, *HRS* Health and Retirement Study, *SHLSE* Survey of Health and Living Status of the Elderly, *ECA* Epidemiologic Catchment Area, *SLAS* Suwon Longitudinal Ageing Study, *KLoSA* Korean Longitudinal Study of Ageing, *BASE* Berlin Ageing Study, *HRS-ss* Health and Retirement Study - subsample of American Indians and Alaska Natives, *MSSA* MacArthur Studies of Successful Ageing, *ELSA* English Longitudinal Study of Ageing, *PATH* Personality and Total Health through Life Study

^1^ *p* values are for continuous network measure; paper also includes *p*-values for categorical network measure, not included in table

Italic text indicates factors that were significantly related

^a^Significant association between socialisation and cognitive measure at baseline

^b^Significant association between socialisation and cognitive measure at follow-up

^c^Significant association between socialisation and cognitive measure at baseline and follow-up

**Table 3:** Characteristics of studies: twin studies

Twin studies examining the impact of social relationships on cognitive function					
Study	Participants	Design	Social Activity Defined /Measure	Cognitive Outcome Measures	Results
Lee (2014) [84] Australia	*N* = 119 pairs of MZ twins Age: 65+ M Age = 71	Discordant MZ twin design	*Social activity:* frequency of engagement in social activities incl. Contact family member, neighbour, friends; talk to neighbour; group activities; church activities; and voluntary work. Adapted from the San Diego Successful Ageing Questionnaire.	*Memory* (composite of Logical Memory Story A, RAVLT, BVRT) Processing speed (composite of TMT-A, Digit Symbol Coding) Verbal fluency (composite of COWAT, Boston Naming Test) Executive function (composite of Digit Span Backward, TMT-B/A, Stroop) Cognitive function (composite of four cognitive domain scores)	Statistically significant association (controls included) between discordance scores for social activity and memory (*p* = 0.007). No other associations found for social activity.
McGue (2007) [82] Denmark (LSADT)	*N* = 70 pairs MZ twins M Age = 77.4 M Age = 75.7	Discordant MZ twin design	*Social activity:* engaging with others (leaving house, party) or mental activity (engaging in a hobby). The Social Activity scale: frequency engaged with others and mental pursuits.	Cognitive Function (MMSE, *composite measure*)	Social activity significantly correlated with initial level of cognitive functioning (*r* = 0.21 for MMSE, 0.44 for cognitive composite score). Social activity was moderately heritable (*r* = 0.36) Significant association between discordance scores and cognitive composite score (*p* < 0.001) but not MMSE (*p* > 0.25).

*MZ* monozygotic, *RAVLT* Rey Auditory Verbal Learning Test, *BVRT* Benson Visual Retention Test, *TMT-A* Trail Making Test-A, *COWAT* Controlled Oral Word Association Test, *TMT-B/A* ratio score of Trail Making Test B/Trail Making Test A, *LSADT* Longitudinal Study of Ageing Danish Twins, *MMSE* Mini Mental Status Examination

### Randomised controlled trials/experimental findings

Three RCTs assessed the impact of social activity on cognitive function with 21 cognitive test measures. Significant improvements were reported for social activity groups from baseline to follow-up on one (out of one) measure of verbal fluency [35]. Compared to a ‘normal community care’ control group, social activity improved performance on one out of three measures of global cognition [34, 35, 57]. Compared to active and placebo controls, non-significant trends for improvement were reported in domains of memory, episodic memory, processing speed, attention and visuospatial processing for engagement in social activity groups [34, 35]. Mortimer et al. also reported significant increases in brain volume in social activity compared to no intervention groups [35]. There were no improvements in performance for social activity groups on one measure of memory, two measures each of attention and processing speed and one measure of executive function [35].

### Observational/longitudinal studies

#### Social activity

Twenty-two observational studies examined the impact of social activity on cognitive function. At baseline, there were significant associations between social activity and higher scores on five out of five measures of global cognition [17, 58–60]; and one out of one measure each of memory and working memory and verbal fluency [60]. At follow-up, significant associations were reported between social activity and performance on 12 out of 14 measures of global cognition [17, 21, 24, 30, 58, 61–70] one (out of one) composite memory measure [71], one (out of one) composite semantic memory measure [66]; four out of nine measures of episodic memory [24, 30, 62, 66, 72, 73]; one out of four measures of executive function [20, 24, 73]; one out of one measure of working memory and visuospatial abilities [66]; three out of five measures of processing speed [30, 37, 62, 66]; and one out of five measures of verbal fluency [24, 62, 71–73]. There were no associations between social activity and scores on two measures of reasoning [30, 73] and one measure of attention [62]. Hsu et al. [64] found that women with unpaid work at baseline were more likely to experience cognitive decline at follow-up; and Small et al. [38] reported that more social activity was related to poorer verbal fluency at follow-up.

#### Social networks

Nine observational studies examined the impact of social networks on cognitive function. Five studies examined network size and frequency of contact with members of the social network together [17, 61, 63, 74, 75] and four studies examined network size alone [21, 32, 67, 76]. At baseline, social network size and frequency of contact were positively correlated with higher scores on measures of global cognition in two studies [17, 32]. At follow-up, six out of nine studies reported a significant association between social network size and frequency of contact with measures of global cognition [17, 21, 61, 67, 74, 75]. Two studies reported no association between social networks and global cognition [32, 63] and one reported no association between social network size and measures of episodic memory, attention, processing speed, or global cognition [76]. Number of social ties was not related to a global cognition in one study [32].

#### Social support

Nine observational studies examined the impact of aspects of social support on cognitive function. At baseline, there was a significant association between emotional support and improved outcomes on four out of five measures of global cognition [22, 32, 58, 76], one measure of reasoning and one measure of processing speed [22]. There were also significant positive associations at baseline between global cognitive function scores and satisfaction with support, negative interactions [76], and conflicts/demands [32]; and a conflicting negative association between baseline global cognition and negative interaction scores [77]. There were no baseline associations reported between instrumental support and scores on three measures of global cognition [22, 32, 76]; or measures of reasoning or processing speed [22]. There were no baseline associations reported between informational support [76] or support to others [32] and outcomes on measures of global cognition.

At follow-up, scores on measures of global cognition were positively correlated with social support [78]. Emotional support was associated with improvements on four out of five global cognition measures [21, 22, 32, 58, 76]. Satisfaction with social support was related to better episodic memory performance, but not attention, processing speed or global cognition [76]. Positive interactions were related to improvements on one out of three measures of episodic memory, one out of three measures of working memory, and two out of three measures of processing speed [79]. One out of three studies reported that negative interactions were related to lower scores on measures of global cognition, episodic memory, semantic memory, working memory, processing speed and visuospatial abilities [76, 77, 79]. Informational support and instrumental support were not related to scores on measures of episodic memory, working memory, attention, processing speed or global cognition in two studies [76, 79].

#### Composite measures of social relationships

Three studies examined the impact of social relationships on cognitive function. At baseline, lower scores on CMSR were associated with poorer verbal fluency and two (out of two) measures of episodic memory [80]. At follow-up, higher scores on CMSR were significantly related to better scores on four out of six measures of episodic memory [27, 80, 81]; and one measure of verbal fluency [80]. There was no reported association between CMSR and global cognition in two studies [27, 81].

### Genetic studies

#### Social activity

Two twin studies examined the impact of social activity on cognitive function. One study reported significant positive correlations between social activity and initial scores on two measures of global cognition [82]. Discordance scores showed significant associations between social activity and improved performance on one measure of memory [83] and on one out of three measures of global cognition [82, 83]. No associations were reported between social activity and outcomes on measures of executive function, verbal fluency or processing speed [83]. Social activity was found to be moderately heritable [82].

## Discussion

Across the four distinct aspects of social relationships, evidence suggests a relationship between (1) social activity and global cognition, overall executive functioning, working memory, visuospatial abilities and processing speed but not episodic memory, verbal fluency, reasoning or attention; (2) social networks and global cognition but not episodic memory, attention or processing speed; (3) social support and global cognition and episodic memory but not attention or processing speed; and (4) CMSR and episodic memory and verbal fluency but not global cognition.

### Social activity

In RCTs, social activity improved global cognition and increased brain volume but did not impact domains of memory, attention, verbal fluency, processing speed or overall executive functioning. Longitudinal associations were reported between social activity and global memory measures, overall executive functioning, working memory, visuospatial abilities, processing speed and global cognition but not episodic memory, verbal fluency, reasoning or attention. Genetic studies showed associations between social activity and memory and global cognition but not overall executive functioning, verbal fluency or processing speed.

Social activity was most consistently associated with improvements on global cognition, as measured by global or composite measures of cognitive function, across all study-types. This replication of results is encouraging, particularly since multiple studies investigated the impact of social activity on global cognition, and suggests that social activity may be useful for promoting brain health in older adults. The included longitudinal and genetic studies also showed that social activity was associated with some (i.e. working memory) but not all (i.e. verbal fluency) executive functioning domains. Although our results fail to support those of Brown et al. [53] who reported that social activity benefitted verbal fluency, the discrepancy may be explained by the number of studies included in each review. Our finding that social activity benefitted working memory supports research on *social working memory* (SWM) that describes working memory as essential for navigating the complexities of the social world [84], and suggests that this relationship is symbiotic. Additional research is required to examine the differential effects of social activity on specific domains of executive functioning, and to determine if social activity interventions can benefit cognitive function or prevent decline.

### Social networks, support and composite scores

Similar to the findings for social activity, the results show that larger social networks and greater levels of social support were associated with improved global cognition. There were also differential effects of the type of social relationships on specific cognitive domains. Social support was associated with benefits to episodic memory but social activity and social networks were not. One explanation for differing effects of social support versus social activity and networks may be the impact that social support has on stress. Social support has been shown to promote resilience against the negative consequences of stress [85] whereas simply engaging in social activities or reporting a larger network of family and friends may not translate to the kind of social-emotional support required to obtain such stress-reducing benefits. Lower levels of stress has been shown to benefit memory and executive performance [47]. Negative interactions on the other hand, may increase stress and have a negative impact on overall cognitive function and on domains of episodic, semantic and working memory, processing speed and visuospatial abilities [76, 77, 79].

Social networks and activity are related concepts and both are structural dimensions of social relationships [85], and individuals who take part in more social activities tend to have a larger social networks (and vice versa). This would explain why social networks and activity appear to similarly impact cognitive domains. Social support on the other hand requires more than a quantity of friends/family and activities, it requires a functional dimension that provides both emotional and instrumental support [86]. This functional dimension is a better predictor of positive health outcomes than the quantitative dimension [87]. This supports ours and prior conclusions that there are distinct effects of different dimensions of social relationships on cognitive abilities [88, 89], and highlights the need for studies that are specifically designed to examine these specific effects. Intervention trials would also help to determine the precise aspects of social relationships that are needed to benefit cognitive function depending on the needs of at-risk older adults.

Scores on the outcome of CMSR were associated with verbal fluency but not global cognition and the findings regarding episodic memory and CMSR were inconsistent. It is difficult to draw any conclusions regarding CMSR because this composite score does not allow the determination of the differential effects of each specific social relationship-type. Future studies would benefit from ensuring consistency and specificity in defining and measuring distinct aspects of social relationships.

### Cognitive decline and social relationships

Overall, the results show that social relationships, as defined in this review, benefits older adults’ cognitive functioning. Changes in the characteristics of social relationships could be a consequence of cognitive decline as opposed to a cause however [25]. The finding that episodic and semantic memory decline are related to a subsequent decline in social activity supports this view [38]. The stigma associated with cognitive decline may lead to social withdrawal [90], failing memory or word-finding difficulties may impede confidence and self-efficacy [91, 92] or poorer cognitive function might result in reduced ability to function socially [25].

Contradictory research has reported that cognitive decline and decline in perceptual speed does not predict decline in social relationships or function [37, 60], and episodic and semantic memory do not predict social activity [71]. In the latter study, while participants with and without cognitive decline both showed decline in social relationships, older individuals engaged in social activities to a lesser extent. Perhaps age may be more influential in affecting social relationships than cognitive decline [93]. Either way, the results demonstrate the complexities of the association between social relationships and older adults’ cognitive function. It is most likely that there is a dual effect, explained by cognitive reserve whereby (1) higher level of engagement promotes positive cognitive outcomes and (2) higher levels of functioning is related to living a more engaged lifestyle [38, 41].

### Limitations and future directions

We found it difficult to identify RCTs that included social relationships as either intervention or active control components. The search terms used may not have identified RCTs that focused on alternative lifestyle factors such as exercise that might have included social relationships as an active control condition, meaning that we may not have included all relevant RCTs. In addition, databases were only searched from the year 2000 to 2017, and Medical Subject Headings (MeSH) were not used meaning that some relevant studies may have been omitted. This was somewhat controlled for through supplemental searches of reference lists of included studies. The more pertinent issue is the fact that there are so few RCTs published that primarily focus on social interventions. This is most likely due to the difficulty in forming appropriate control conditions, although socially isolated older adults are not uncommon—however, they are, by their very nature, a difficult group to access for research. An RCT that recruited socially isolated older adults into four conditions: (1) social activity, (2) social networks; (3) social support; (4) wait-list control and examined outcomes on cognitive and social functioning measures would provide important insights.

The heterogeneity in definitions and measures of social relationships and cognitive function resulted in difficulties collating research evidence in a meaningful way and precluded us from conducting a meta-analysis. To promote homogeneity, future research could use the categorisation and definitions of social relationships as outlined in this review. Studies would benefit from an agreed appropriate battery of cognitive tests to be used when examining each distinct aspect of social relationships; for example, measures of episodic memory might be more important when investigating social support than networks or activity, and studies would benefit from including measures of global cognition and working memory for all social relationship-types. Systematic reviews consistently call for standardisation of tests measures to improve replicability and test the reliability of individual study findings, and yet standardisation and replication remain largely absent from this literature. Future RCT and longitudinal studies need to replicate prior studies with a view to strengthening existing evidence and determining the exact nature of the association between social relationships and cognitive function.

Exploring the impact of social relationships as distinct from cognitive, leisure or physical domains of activity is questionable, since all social behaviour includes aspects of these three domains of activity and it is not possible to isolate purely social factors. Many leisure activities have a physical (dancing, walking) or cognitive (playing chess) element which impact on cognitive processes and may confound measurement of social behaviour. To design effective RCTs, researchers could design interventions that include social activities, networks and support, and ensure these are clearly defined and consistently used across studies to improve comparability of results. It would also be helpful to avoid incorporating clear physical exercise or cognitive stimulation in social interventions which may confound results. Future research might also consider the impact of technology, internet and social media on social relationships, particularly feelings of social support.

Loneliness was not considered in the current review. While previous meta-analyses and reviews have investigated loneliness and social isolation together [94, 95], with regards to other outcomes, loneliness is often experienced as a psychological phenomenon which is not entirely contingent on social engagement but instead at least partly attributable to factors such as maladaptive social cognitions [96] and feelings of physical security [97]. As such, loneliness may not be suitable for inclusion in discussions concerning social relationships per se, although a detailed account of social support may describe loneliness where perceived social support is lacking.

## Conclusions

Researchers suggest that the size of the association between social relationships and cognition is similar to that of age and cognition [63, 72]. Our review adds to and supports existing research and findings show that there is an association between social relationships and the cognitive functioning of healthy older adults, although the specific nature of this association remains unclear. This review is novel in its examination of different aspects of social relationships, namely social activity, social networks, social support, and CMSR, and the differential effects these factors have on cognitive functioning. Evidence was most consistent in favour of a relationship between the distinct forms of social relationships and global cognition and working memory. It is important to reiterate the necessity to define social relationships more clearly to achieve homogeneity across studies [14, 40, 98]. Future research needs to achieve consistency in social and cognitive definitions and measures, replication of prior correlational findings, and the design of appropriate RCTs to provide a more thorough and meaningful investigation of the impact of social relationships on the cognitive functioning of healthy older adults.

## Additional files

Additional file 1: Search results. Table of search terms and results as used in the systematic literature search. (DOCX 19 kb)

Additional file 2: Excluded studies. Table of studies excluded from the systematic review. (DOCX 28 kb)

Additional file 3: Risk of bias in RCTs. Table including information regarding the risk of bias assessment conducted on RCTs. (DOCX 18 kb)

Additional file 4: STROBE quality assessment. Table including information of the quality assessment conducted on all observational studies. (XLSX 16 kb)

## Abbreviations

ADAS-Cog: Alzheimer’s Disease Assessment Scale-Cognition
AVLT: Auditory Verbal Learning Test
BASE: Berlin Ageing Study
BVRT: Benson Visual Retention Test
Cantab: Cambridge Tests of Cognitive Function
CAPE: Clifton Assessment Procedure for the Elderly
CHAP: Chicago Health and Ageing Project
CMSR: Composite Measures of Social Relationships
COWAT: Controlled Oral Word Association Test
CSHA: Canadian Study of Health and Ageing
CSID: Community Screening Instrument for Dementia
CVLT: California Verbal Learning Test
DSB: Digit span backward
DSF: Digit span forward
DSS: Digit symbol substitution
ECA: Epidemiologic catchment area
ELSA: English Longitudinal Study of Ageing
EPESE: Established Populations for Epidemiologic Studies of the Elderly
ETS-CA: Educational Testing Service Kit-Controlled Associations Test
FU: Follow-up
HRS: Health and Retirement Study
HRS-ss: Health and Retirement Study—subsample of American Indians and Alaska Natives
HVLT: Hopkins Verbal Learning Test
KLoSA: Korean Longitudinal Study of Ageing
LAS: Longitudinal Ageing Study
LASA: Longitudinal Ageing Study Amsterdam
LSADT: Longitudinal Study of Ageing Danish Twins
M Age: Mean age
MAAS: Maastricht Ageing Study
MDRS: Mattis Dementia Rating Scale
MMSE: Mini Mental Status Examination
MSSA: MacArthur Studies of Successful Ageing
MZ: Monozygotic
PATH: Personality and Total Health Through Life Study
RAVLT: Rey Auditory Verbal Learning Test
RCTs: Randomised controlled trials
Rey CFT: Rey Complex Figure Task
SDMT: Symbol-Digit Modalities Test
SHARE: Survey of Health Ageing and Retirement in Europe
SHLSE: Survey of Health and Living Status of the Elderly
SLAS: Suwon Longitudinal Ageing Study
SPMSQ: Short Portable Mental Status Questionnaire
STROBE: Strengthening the Reporting of Observational Studies in Epidemiology
TICS: Telephone Interview for Cognitive Status
TLSA: Taiwan Longitudinal Study on Ageing
TMT: Trail Making Test
TMT-A: Trail Making Test-A
TMT-B/A: Ratio Score of Trail Making Test B/Trail Making Test A
VLS: Victoria Longitudinal Study
WAIS-R: Wechsler Adult Intelligence Scale-Revised
WMS: Wechsler Memory Scale
