# Supplementary material for: The impact of social activities, social networks, social support and social relationships on the cognitive functioning of healthy older adults: a systematic review
Source: Syst Rev. 2017 Dec 19;6:259. doi: 10.1186/s13643-017-0632-2 (PMC5735742; doi:10.1186/s13643-017-0632-2)
Supplement: Supplementary file 1 — Search results. Table of search terms and results as used in the systematic literature search. (DOCX 19 kb) [file 13643_2017_632_MOESM1_ESM.docx]

| **Search Terms – Socialisation and Cognition** | **Articles Found** | | **Full Text Screened** |
| --- | --- | --- | --- |
| A1A  (“social” OR “social engagement” OR “leisure” OR “social intervention” OR “leisure intervention” OR “isolation” OR “social network” OR “relationships”) AND (“cognition” OR “cognitive performance” OR “cognitive decline” OR “cognitive function ” OR “cognitive processes” OR “cognitive ageing” OR “mental” ) AND (“healthy elderly” OR “elderly” OR “older adults” OR “old adults” OR “aging” OR “ageing”) | 2000-2015 | 6981 | 21 |
|  |  |  |  |
| A2A  (“social” OR “social engagement” OR “leisure” OR “social intervention” OR “leisure intervention” OR “isolation” OR “social network” OR “relationships”)AND (“intelligence” OR “executive function” OR “executive control” OR “processing” OR “reaction time” OR “memory” OR “perception” OR “attention”) AND (“healthy elderly” OR “elderly” OR “older adults” OR “old adults” OR “aging” OR “ageing” ) | 2000-2015 | 4954 | 45 |
| B1A  (“social ties” OR “social activities” OR “social activity” OR “leisure activities” OR “leisure activity” OR “social relations” OR “social engagement” OR “recreational” )AND (“cognition” OR “cognitive performance” OR “cognitive decline” OR “cognitive function ” OR “cognitive processes” OR “cognitive ageing” OR “mental” ) AND (“healthy elderly” OR “elderly” OR “older adults” OR “old adults” OR “aging” OR “ageing”) | 2000-2015 | 579 | 1 |
| B2A  (“social ties” OR “social activities” OR “social activity” OR “leisure activities” OR “leisure activity” OR “social relations” OR “social engagement” OR “recreational” ) AND (“intelligence” OR “executive function” OR “executive control” OR “processing” OR “reaction time” OR “memory” OR “perception” OR “attention” ) AND ( “healthy elderly” OR “elderly” OR “older adults” OR “old adults” OR “aging” OR “ageing”) | 2000-2015 | 294 | 3 |
| C1A  “social isolation” OR “social mobility” OR “social stimulation” OR “social control” OR “social control group” OR “social support” OR “social group” OR “socialisation” OR “social factors”) AND (“cognition” OR “cognitive performance” OR “cognitive decline” OR “cognitive function ” OR “cognitive processes” OR “cognitive ageing” OR “mental” ) AND (“healthy elderly” OR “elderly” OR “older adults” OR “old adults” OR “aging” OR “ageing”) | 2000-2015 | 1519 | 5 |
| C2A  ( “social isolation” OR “social mobility” OR “social stimulation” OR “social control” OR “social control group” OR “social support” OR “social group” OR “socialisation” OR “social factors”) AND ( “intelligence” OR “executive function” OR “executive control” OR “processing” OR “reaction time” OR “memory” OR “perception” OR “attention” ) AND ( “healthy elderly” OR “elderly” OR “older adults” OR “old adults” OR “aging” OR “ageing”) | 2000-2015 | 769 | 0 |

| **Search Update January 2017** | | | |
| --- | --- | --- | --- |
| **Search Terms** |  | **Found** | **Full Text Screened** |
| (“social” OR “social engagement” OR “leisure” OR “social intervention” OR “leisure intervention” OR “isolation” OR “social network” OR “relationships”) AND (“cognition” OR “cognitive performance” OR “cognitive decline” OR “cognitive function ” OR “cognitive processes” OR “cognitive ageing” OR “mental” ) AND (“healthy elderly” OR “elderly” OR “older adults” OR “old adults” OR “aging” OR “ageing”) | Jan 2014-Jan 2017 | 1869 | 2 |
| "social" AND "cognitive function" AND "older" AND "adults" | Jan 2014-Jan 2017 | 185 | 12 |
| (“social” OR “social engagement” OR “leisure” OR “isolation” OR “social network” OR “relationships”) AND (“cognition” OR “cognitive performance” OR “cognitive decline” OR “cognitive function”) AND (“elderly” OR “older adults” OR “aging”) | Jan 2014-Jan 2017 | 809 | 18 |
