# Supplementary material for: The impact of social activities, social networks, social support and social relationships on the cognitive functioning of healthy older adults: a systematic review
Source: Syst Rev. 2017 Dec 19;6:259. doi: 10.1186/s13643-017-0632-2 (PMC5735742; doi:10.1186/s13643-017-0632-2)
Supplement: Supplementary file 2 — Excluded studies. Table of studies excluded from the systematic review. (DOCX 28 kb) [file 13643_2017_632_MOESM2_ESM.docx]

| **Excluded Studies (based on abstracts and titles)** | |
| --- | --- |
| **Reference** | **Reason for Exclusion** |
| 1. Andel, R., Silverstein, M., Kåreholt, I. The role of midlife occupational complexity and leisure activity in late-life cognition. J Gerontol B Psychol Sci Soc Sci. 2014 Sep; 70(2): 314-321. Epub 2014 Sep 4 PubMed PMID: 25190210 | <50 years |
| 1. Andrew MK, Rockwood K. Social vulnerability predicts cognitive decline in a prospective cohort of older Canadians. Alzheimers Dement. 2010 Jul; 6 (4):319-325.e1. doi: 10.1016/j.jalz.2009.11.001. | Duplicate |
| 1. Beauchet, O., Launay, C.P., Merjagnan, C., Kabeshova, A., Annweiler, C. Quantified self and comprehensive geriatric assessment: older adults are able to evaluate their own health and functional status. PLoS One. 2014 Jun; 26 9(6). | Outcome not relevant |
| 1. Belley, A.M., Parisien, M., Nour, K., Bier, N., Ferland, G., Guay, D., Popov, P., Laforest, S. An ecological perspective on the determinants of the cognitive vitality of seniors. Can J Aging. 2013 Sep; 32(3): 240-249. | Inappropriate design |
| 1. Benloucif S, Orbeta L, Ortiz R, Janssen I, Finkel SI, Bleiberg J, Zee PC.   Morning or evening activity improves neuropsychological performance and  subjective sleep quality in older adults. Sleep. 2004 Dec 15;27(8):1542-51. | Outcome not relevant |
| 1. Bielak, A.A., Cherbuin, N., Bunce, D., Anstey, K.J. Preserved differentiation between physical activity and cognitive performance across young, middle, and older adulthood over 8 years. J Gerontol B Psychol Sci Soc Sci. 2014 Jul; 69(4): 523-32. | <50 years |
| 1. Brown CL, Robitaille A, Zelinski EM, Dixon RA, Hofer SM, Piccinin AM.   Cognitive activity mediates the association between social activity and cognitive  performance: A longitudinal study. Psychol Aging. 2016 Dec;31(8):831-846. | Inappropriate design |
| 1. Buz, J., Sanchez, M., Levenson, M.R., Aldwin, C.M. Aging and social networks in Spain: the importance of pubs and churches. Int J Aging Hum Dev. 2014; 78(1): 23-46 PubMed PMID: 24669508 | Outcome not relevant |
| 1. Carlson MC, Saczynski JS, Rebok GW, Seeman T, Glass TA, McGill S, Tielsch J, Frick KD, Hill J, Fried LP. Exploring the effects of an "everyday" activity program on executive function and memory in older adults: Experience Corps.   Gerontologist. 2008 Dec;48(6):793-801. | Inappropriate design |
| 1. Chiu, Y.C., Huang, C.Y., Kolanowski, A.M., Huang, H.L., Shyu, Y.L., Lee, S.H., Lin, C.R., Hsu, W.C. The effects of participation in leisure activities on neuropsychiatric symptoms of persons with cognitive impairment: a cross-sectional study. Int J Nurs Stud. 2013 Oct; 50(10):1314-25. Epub 2013 Feb 11 PubMed PMID: 23411009 | Inappropriate sample |
| 1. Choi Y, Park S, Cho KH, Chun SY, Park EC. A change in social activity affect   cognitive function in middle-aged and older Koreans: analysis of a Korean  longitudinal study on aging (2006-2012). Int J Geriatr Psychiatry. 2016  Aug;31(8):912-9. doi: 10.1002/gps.4408. PubMed PMID: 26833847. | <50 years |
| 1. Conroy RM, Golden J, Jeffares I, O'Neill D, McGee H. Boredom-proneness,   loneliness, social engagement and depression and their association with cognitive  function in older people: a population study. Psychol Health Med. 2010  Aug;15(4):463-73. | Cross-sectional |
| 1. Dikmen, S.S., Bauer, P.J., Weintraub, S., Mungas, D., Slotkin, J., Beaumont, J.L., Gershon, R., Temkin, N.R., Heaton, R.K. Measuring episodic memory across the lifespan: NIH Toolbox Picture Sequence Memory Test. J Int Neuropsychol Soc. 2014 Jul; 20(6): 611-619. Epub 2014 Jun 24 PMID: 24960230 | Outcome not relevant |
| 1. DiNapoli EA, Wu B, Scogin F. Social isolation and cognitive function in   Appalachian older adults. Res Aging. 2014 Mar;36(2):161-79. doi:  10.1177/0164027512470704. | Cross-sectional |
| 1. Dodge, H.H,, Ybarra, O., Kaye, J.A. Tools for advancing research into social networks and cognitive function in older adults. Int Psychogeriatr. 2014 April; 26(4): 533–539. Epub 2013 Oct 23 PubMed PMID: 24152936 | Inappropriate design |
| 1. Dregan, A., Gulliford, M.C. Leisure-time physical activity over the life course and cognitive functioning in late mid-adult years: a cohort-based investigation. Psychol Med. 2013 Nov; 43(11): 2447-2458. Epub 2013 Mar 12 PubMed PMID: 23480851 | <50 years |
| 1. Franco-Martín, M., Parra-Vidales, E., González-Palau, F., Bernate-Navarro, M., Solis, A. The influence of physical exercise in the prevention of cognitive deterioration in the elderly: a systematic review. Rev Neurol. 2013 Jun; 56(11), 545-554. PubMed PMID: 23703056 | Outcome not relevant |
| 1. Fratiglioni, L., Wang, H.-X., Ericsson, K., Maytan, M., & Winblad, B. (2000). Influence of social network on occurrence of dementia: a community-based longitudinal study. *The Lancet, 355*(9212), 1315-1319. | Inappropriate sample |
| 1. Fujiwara, Y., Suzuki, H., Kawai, H., Hirano, H., Yoshida, H., Kojima, M., Ihara, K., Obuchi, S. Physical and sociopsychological characteristics of older community residents with mild cognitive impairment as assessed by the Japanese version of the Montreal Cognitive Assessment. J Geriatr Psychiatry Neurol. 2013 Dec; 26(4): 209-220. | Inappropriate sample |
| 1. Gallucci M, Antuono P, Ongaro F, Forloni PL, Albani D, Amici GP, Regini C.   Physical activity, socialization and reading in the elderly over the age of seventy: what is the relation with cognitive decline? Evidence from "The Treviso Longeva (TRELONG) study". Arch Gerontol Geriatr. 2009 May-Jun;48(3):284-6. | Cross-sectional |
| 1. Ghisletta, P., Bickel, J.F., Lövdén, M. Does activity engagement protect against cognitive decline in old age? Methodological and analytical considerations. Journal of Gerontology: Psychological Sciences, 2006 Sep; 61(5): 253–261. PubMed PMID: 16960228 | Inappropriate design |
| 1. Grande, G., Vanacore, N., Maggiore, L., Cucumo, V., Ghiretti, R., Galimberti, D., Scarpini, E., Mariani, C., Clerici, F. Physical activity reduces the risk of dementia in mild cognitive impairment subjects: a cohort study. [J](http://www.ncbi.nlm.nih.gov/pubmed/24296815) Alzheimers Dis. 2014; 39(4): 833-9. | Inappropriate sample |
| 1. Green AF, Rebok G, Lyketsos CG. Influence of social network characteristics on   cognition and functional status with aging. Int J Geriatr Psychiatry. 2008  Sep;23(9):972-8. doi: 10.1002/gps.2023. | <50 years |
| 1. Hamer, M., Stamatakis, E. Prospective study of sedentary behavior, risk of depression, and cognitive impairment. Med Sci Sports Exerc. 2014 April; 46(4): 718-723. PMID: 24121248 | Inappropriate sample |
| 1. Harmell, A.L., Jeste, D., Depp, C. Strategies for successful aging: a research update. Curr Psychiatry Rep. 2014 Oct; 16(10): 476. PubMed PMID: 25135776 | Review |
| 1. Haslam, C., Alexander Haslam, S., Knight, C., Gleibs, I., Ysseldyk, R., McCloskey, L.G. We can work it out: Group decision-making builds social identity and enhances the cognitive performance of care residents. Social identification moderates cognitive health and well-being following story- and song-based reminiscence, Aging & Mental Health. 2014 Feb; 18(4): 425-434. Epub 2012 Dec 17. PubMed PMID: 24387094 | Inappropriate sample |
| 1. Haslam C, Cruwys T, Milne M, Kan CH, Haslam SA. Group Ties Protect Cognitive Health by Promoting Social Identification and Social Support. J Aging Health. 2016 Mar;28(2):244-66. doi: 10.1177/0898264315589578. | Cross-sectional |
| 1. Hindle, J.V., Martyr, A., Clare, L. Cognitive reserve in Parkinson's disease: a systematic review and meta-analysis. Parkinsonism Relat Disord. 2014 Jan; 20(1):1-7. Epub 2013 Aug 29 PubMed PMID: 24034887 | Inappropriate sample |
| 1. Howrey BT, Raji MA, Masel MM, Peek MK. Stability in Cognitive Function Over 18 Years: Prevalence and Predictors among Older Mexican Americans. Curr Alzheimer Res. 2015;12(7):614-21. | Outcome not relevant |
| 1. Hoogendijk EO, Deeg DJ, Poppelaars J, van der Horst M, Broese van Groenou MI, Comijs HC, Pasman HR, van Schoor NM, Suanet B, Thomése F, van Tilburg TG, Visser M, Huisman M. The Longitudinal Aging Study Amsterdam: cohort update 2016 and major findings. Eur J Epidemiol. 2016 Sep;31(9):927-45. doi:10.1007/s10654-016-0192-0. | Duplicate |
| 1. Hughes, T.F., Flatt, J.D., Fu, B., Chang, C.C., Ganguli, M. Engagement in social activities and progression from mild to severe cognitive impairment: the MYHAT study. Int Psychogeriatr. 2013 April; 25(4):587-595. Epub 2012 Dec 21 PubMed PMID: 23257280 | Inappropriate sample |
| 1. Jonaitis, E., La Rue, A., Mueller, K.D., Koscik, R.L., Hermann, B., Sager, M.A. Cognitive activities and cognitive performance in middle-aged adults at risk for Alzheimer's disease. Psychol Aging. 2013 Dec; 28(4):1004-1014. PubMed PMID: 2436440 | Outcome not relevant |
| 1. Jopp DS, Park MK, Lehrfeld J, Paggi ME. Physical, cognitive, social and mental   health in near-centenarians and centenarians living in New York City: findings  from the Fordham Centenarian Study. BMC Geriatr. 2016 Jan 5;16:1. doi:  10.1186/s12877-015-0167-0. | Cross-sectional |
| 1. Kåreholt I, Lennartsson C, Gatz M, Parker MG. Baseline leisure time activity and cognition more than two decades later. Int J Geriatr Psychiatry. 2011   Jan; 26(1):65-74. doi: 10.1002/gps.2490. | <50 years |
| 1. Kats D, Patel MD, Palta P, Meyer ML, Gross AL, Whitsel EA, Knopman D, Alonso A, Mosley TH, Heiss G. Social support and cognition in a community-based cohort: the Atherosclerosis Risk in Communities (ARIC) study. Age Ageing. 2016Jul;45(4):475-80. doi: 10.1093/ageing/afw060. | Inappropriate sample |
| 1. Kim D, Arai H, Kim S. Social activities are associated with cognitive decline   in older Koreans. Geriatr Gerontol Int. 2016 Sep 26. doi: 10.1111/ggi.12861.  [Epub ahead of print] PubMed PMID: 27667726. | Cross-sectional |
| 1. Kotwal AA, Kim J, Waite L, Dale W. Social Function and Cognitive Status:   Results from a US Nationally Representative Survey of Older Adults. J Gen Intern Med. 2016 Aug;31(8):854-62. doi: 10.1007/s11606-016-3696-0. | Cross-sectional |
| 1. Krueger KR, Wilson RS, Kamenetsky JM, Barnes LL, Bienias JL, Bennett DA.   Social engagement and cognitive function in old age. Exp Aging Res. 2009  Jan-Mar;35(1):45-60. | Cross-sectional |
| 1. Kuiper JS, Oude Voshaar RC, Zuidema SU, Stolk RP, Zuidersma M, Smidt N. The relationship between social functioning and subjective memory complaints in older persons: a population-based longitudinal cohort study. Int J Geriatr Psychiatry. 2016 Aug 22. doi: 10.1002/gps.4567. [Epub ahead of print] PubMed PMID: 27546724. | Outcome not relevant |
| 1. Küster OC, Fissler P, Laptinskaya D, Thurm F, Scharpf A, Woll A, Kolassa S,   Kramer AF, Elbert T, von Arnim CA, Kolassa IT. Cognitive change is more  positively associated with an active lifestyle than with training interventions  in older adults at risk of dementia: a controlled interventional clinical trial.  BMC Psychiatry. 2016 Sep 8;16(1):315. | Inappropriate design |
| 1. Lam LC, Ong PA, Dikot Y, Sofiatin Y, Wang H, Zhao M, Li W, Dominguez J,   Natividad B, Yusoff S, Fu JL, Senanarong V, Fung AW, Lai K. Intellectual and  physical activities, but not social activities, are associated with better global  cognition: a multi-site evaluation of the cognition and lifestyle activity study  for seniors in Asia (CLASSA). Age Ageing. 2015 Sep;44(5):835-40. doi:  10.1093/ageing/afv099. | Cross-sectional |
| 1. Langlois, F., Vu, T.T., Chassé, K., Dupuis, G., Kergoat, M.J., Bherer, L. Benefits of physical exercise training on cognition and quality of life in frail older adults. J Gerontol B Psychol Sci Soc Sci. 2013 MAy; 68(3): 400-404. Epub 2012 Aug 28 PubMed PMID: 22929394 | Outcome not relevant |
| 1. Lee, J., Shih, R., Feeney, K., Langa, K.M. Gender disparity in late-life cognitive functioning in India: findings from the longitudinal aging study in India. Journals of Gerontology, Series B: Psychological Sciences and Social Sciences. 2014 Jul; 69(4), 514–522. PubMed PMID :24622150 | <50 years |
| 1. Liao, J., Head, J., Kumari, M., Stansfeld, S., Kivimaki, M., Singh-Manoux, A., Brunner, E.J. (2014). Negative aspects of close relationships as risk factors for cognitive aging. Am J Epidemiol. 2014 Dec; 180(11): 1118-1125. Epub 2014 Oct 22 | <50 years |
| 1. Liao J, Muniz-Terrera G, Head J, Brunner EJ. Dynamic Longitudinal Associations Between Social Support and Cognitive Function: A Prospective Investigation of the Directionality of Associations. J Gerontol B Psychol Sci Soc Sci. 2016 Nov 1. pii: gbw135. [Epub ahead of print] PubMed PMID: 27803025. | No full text |
| 1. Litwin H, Stoeckel KJ. Social Network, Activity Participation, and Cognition:   A Complex Relationship. Res Aging. 2016 Jan;38(1):76-97. doi:  10.1177/0164027515581422. | Cross-sectional |
| 1. Mahendra, N., Arkin, S. Effects of four years of exercise, language, and social interventions on Alzheimer discourse. Journal of Communication Disorders. 2003 Sep-Oct; 36: 395–422. | Inappropriate sample |
| 1. McGue, M., Skytthe, A., Christensen, K.. (2014). The nature of behavioural correlates of healthy ageing: a twin study of lifestyle in mid to late life. Int J Epidemiol. 2014 Jun; 43(3): 775-782. | <50 years |
| 1. McHugh Power J, Tang J, Lawlor B, Kenny RA, Kee F. Mediators of the   relationship between social activities and cognitive function among older Irish  adults: results from the Irish longitudinal study on ageing. Aging Ment Health.  2016 Sep 27:1-6. [Epub ahead of print] PubMed PMID: 27676290. | Inappropriate design |
| 1. Milán-Calenti, J.C., Sánchez, A., Lorenzo-Lopez, L., Cao, R., Maseda, A. Influence of social support on older adults with cognitive impairment, depressive symptoms, or both coexisting. The International Journal of Aging and Human Development. 2013; 76: 199-214. | Inappropriate sample |
| 1. Mortensen, E.L., Flensborg-Madsen, T., Molbo, D., Christensen, U., Osler, M., Avlund, K., Lund, R. Personality in late midlife: associations with demographic factors and cognitive ability. J Aging Health. 2014 Feb; 26(1): 21-36. | <50 years |
| 1. Myhre JW, Mehl MR, Glisky EL. Cognitive Benefits of Online Social Networking for Healthy Older Adults. J Gerontol B Psychol Sci Soc Sci. 2016 Mar 16. pii: gbw025. [Epub ahead of print] PubMed PMID: 26984523. | Inappropriate design |
| 1. Nouchi, R., Taki, Y., Takeuchi, H., Sekiguchi, A., Hashizume, H., Nozawa, T., Nouchi, H., Kawashima, R. Four weeks of combination exercise training improved executive functions, episodic memory, and processing speed in healthy elderly people: evidence from a randomized controlled trial. Age (Dordr), 2014 April; 36(2): 787-99. | Outcome not relevant |
| 1. OCTO Twin Studies | Outcome not relevant or pre 2000 |
| 1. Pillemer SC, Holtzer R. The differential relationships of dimensions of   perceived social support with cognitive function among older adults. Aging Ment  Health. 2016 Jul;20(7):727-35. doi: 10.1080/13607863.2015.1033683. | Cross-sectional |
| 1. Richards M, Hardy R, Wadsworth ME. Does active leisure protect cognition? Evidence from a national birth cohort. Soc Sci Med. 2003; 56(4):785–792. | <50 years |
| 1. Sims RC, Hosey M, Levy SA, Whitfield KE, Katzel LI, Waldstein SR. Distinct   functions of social support and cognitive function among older adults. Exp Aging Res. 2014;40(1):40-59. doi: 10.1080/0361073X.2014.857551. | Cross-sectional |
| 1. Sörman DE, Sundström A, Rönnlund M, Adolfsson R, Nilsson LG. Leisure activity   in old age and risk of dementia: a 15-year prospective study. J Gerontol B  Psychol Sci Soc Sci. 2014 Jul;69(4):493-501. | Outcome not relevant |
| 1. Stine-Morrow EA, Parisi JM, Morrow DG, Greene J, Park DC. An engagement model of cognitive optimization through adulthood. J Gerontol B Psychol Sci Soc Sci.2007 Jun;62 Spec No 1:62-9. | Inappropriate design |
| 1. Tilvis, R.S., et al. (2004). Predictors of cognitive decline and mortality of aged people over a 10-year period. | Outcome not relevant |
| 1. Tun PA, Miller-Martinez D, Lachman ME, Seeman T. Social strain and executive function across the lifespan: the dark (and light) sides of social engagement. Neuropsychol Dev Cogn B Aging Neuropsychol Cogn. 2013;20(3):320-38. doi:10.1080/13825585.2012.707173. | <50 years |
| 1. Wang JY, Zhou DH, Li J, Zhang M, Deng J, Tang M, Gao C, Li J, Lian Y, Chen M. Leisure activity and risk of cognitive impairment: the Chongqing aging study.   Neurology. 2006 Mar 28;66(6):911-3. | Inappropriate design |
| 1. Wang C, Zhu J, Cai Y, Cui D, Wang Q, Mao Z. Community-Based Study of the   Relationship Between Social Capital and Cognitive Function in Wuhan, China. Asia Pac J Public Health. 2016 Nov;28(8):717-724. | Cross-sectional |
| 1. Wu F, Guo Y, Zheng Y, Ma W, Kowal P, Chatterji S, Wang L. Social-Economic   Status and Cognitive Performance among Chinese Aged 50 Years and Older. PLoS One. 2016 Nov 18;11(11):e0166986. doi: 10.1371/journal.pone.0166986. | Cross-sectional |
| 1. Ybarra O, Burnstein E, Winkielman P, Keller MC, Manis M, Chan E, Rodriguez J. Mental exercising through simple socializing: social interaction promotes general cognitive functioning. Pers Soc Psychol Bull. 2008 Feb;34(2):248-59 | <50 years |
| 1. Zamora-Macorra M, de Castro EF, Ávila-Funes JA, Manrique-Espinoza BS,   López-Ridaura R, Sosa-Ortiz AL, Shields PL, Del Campo DS. The association between social support and cognitive function in Mexican adults aged 50 and older. Arch Gerontol Geriatr. 2017 Jan - Feb; 68: 113-118. doi: 0.1016/j.archger.2016.10.005. | Cross-sectional |
| 1. Zhang, Y., Shi, Z., Liu, M., Liu, S., Yue, W., Liu, S., Xiang, L., Lu, H., Liu, P., Wisniewski, T., Wang, J., Ji, Y. (2014). Prevalence of cognitive impairment no dementia in a rural area of Northern China. Neuroepidemiology. 2014; 42(4): 197–203. | Outcome not relevant |
| 1. Zhong BL, Chen SL, Tu X, Conwell Y. Loneliness and Cognitive Function in Older Adults: Findings From the Chinese Longitudinal Healthy Longevity Survey. J Gerontol B Psychol Sci Soc Sci. 2017 Jan;72(1):120-128. | Outcome not relevant |
