# Supplementary material for: The impact of social activities, social networks, social support and social relationships on the cognitive functioning of healthy older adults: a systematic review
Source: Syst Rev. 2017 Dec 19;6:259. doi: 10.1186/s13643-017-0632-2 (PMC5735742; doi:10.1186/s13643-017-0632-2)
Supplement: Supplementary file 3 — Risk of bias in RCTs. Table including information regarding the risk of bias assessment conducted on RCTs. (DOCX 18 kb) [file 13643_2017_632_MOESM3_ESM.docx]

| **Mortimer (2012)** | | | | | |
| --- | --- | --- | --- | --- | --- |
| **Risk of Bias** | **Sequence generation** | **Allocation concealment** | **Blinding** | **Incomplete outcome data** | **Selective outcome reporting** |
| **High** |  | Unlikely that allocation was concealed. | Participants were informed of all interventions as part of consent procedure. Could be argued that control participants received slight social intervention through phone calls. |  |  |
| **Moderate** |  |  |  | Similar attrition rates between groups and groups still balanced for gender, and Dementia Rating Scale scores. |  |
| **Low** |  |  |  |  | All outcomes reported. |
| **Unclear** | Used ‘stratified randomization’ to ensure approximately equal sex balance. No information on how this was done. |  |  |  |  |
| **Park (2014)** | | | | | |
| **Risk of Bias** | **Sequence generation** | **Allocation concealment** | **Blinding** | **Incomplete outcome data** | **Selective outcome reporting** |
| **High** | Allocation stated as randomised but participants able to exclude themselves from their least desired intervention. |  |  |  |  |
| **Moderate** |  |  | Testers were blind to participant condition but participants aware of all conditions (but not aware that a placebo condition existed). | Reasons for dropout recorded. Attrition rates between groups assessed as not directly related to group assignment. |  |
| **Low** |  |  |  |  | All outcomes reported. (no-treatment condition excluded from analyses as its function was to investigate test-retest reliability) |
| **Unclear** |  | Allocation procedure not described. |  |  |  |
| **Pitkala (2011)** | | | | | |
| **Risk of Bias** | **Sequence generation** | **Allocation concealment** | **Blinding** | **Incomplete outcome data** | **Selective outcome reporting** |
| **High** | Participants divided into 3 groups related to preferences of activities. Within each group, participants were further divided into a randomised intervention and control group. Some participants from outside the sample originally invited self-selected by seeking access to group psychotherapy. |  |  |  |  |
| **Moderate** |  | Random assignment into intervention groups of 16 based on preference and further into control and intervention groups was conducted by an allocator using a randomisation programme and who had access to only participant names. | Testers did not participate in or discuss interventions with participants but were not blind to conditions. | Clinical outcome variables were analysed by intention-to-treat principle, with multiple imputation for missing data. |  |
| **Low** |  |  |  |  | All outcomes reported. |
| **Unclear** |  |  |  |  |  |
|  |  |  |  |  |  |
|  |  |  |  |  |  |

**Scoring:** A higher score indicates greater risk of bias; High = 4; Medium = 3; Unclear = 2; Low = 1. High RoB = 20; Medium RoB = 15; Unclear = 10; Low = 5.

Mortimer scores 2 high (8), 1 medium (3), 1 unclear (2) and 1 low (1). **Total = 14**

Park scores 1 high (4), 2 medium (6), 1 unclear (2) and 1 low (1). **Total = 13**

Pitkala scores 1 high (4), 3 medium (9), 0 unclear (0), 1 low (1). **Total = 14**
